# Effect of Substrate Moisture Content on the Growth of an Exotic Species, *Myriophyllum aquaticum*

**DOI:** 10.3390/plants15111742

**Published:** 2026-06-04

**Authors:** Mingkai Leng, Xiaodong Wu, Xing Wang, Xuguang Ge, Fan Xun, Xinhui Yu, Haoran Liu, Haoyue Li, Xin Mou

**Affiliations:** 1 College of Urban and Environmental Sciences, Hubei Normal University, Huangshi 435002, China; lmk0361h@163.com (M.L.); 2024x07051106@stu.hbnu.edu.cn (X.W.); gxg76@hbnu.edu.cn (X.G.); xunfan130@163.com (F.X.); yuxinhui_1998@163.com (X.Y.); 15572435513@163.com (H.L.); 2024x07051108@stu.hbnu.edu.cn (H.L.); muxin021126@163.com (X.M.); 2 Wanghu Lake Wetland Ecosystem Field Scientific Observation and Research Station, Hubei Normal University, Huangshi 435002, China; 3 Huangshi Key Laboratory of Soil Pollution and Control, Huangshi 435002, China

**Keywords:** *Myriophyllum aquaticum*, substrate moisture content, exotic species, submerged plants

## Abstract

In this study, we investigated how substrate moisture content affects the growth performance and adaptive responses of *Myriophyllum aquaticum*. Through a controlled simulation experiment, we systematically analyzed the morphological characteristics and physiological responses of plants under five moisture levels: 0–15%, 15–30%, 30–45%, 45–60%, and 60–75%. The results indicate that optimal growth of *M. aquaticum* occurred at a substrate moisture content of 60–75%, with significant increases in plant height, branching ability, and biomass. A drought acclimation response was triggered at moisture levels ≤45%, characterized by shortened root length, increased total senescent internode length, biomass allocation shift toward aboveground parts, decreased chlorophyll a content, and elevated accumulation of malondialdehyde. Plants died at moisture levels ≤15%. However, they survived at 15–30% moisture, although their biomass continued to decline. A key finding was that under conditions where the sediment surface lacked water but the substrate moisture remained at 60–75%, plants achieved efficient water utilization and canopy reconstruction through rapid root extension and stem node proliferation, and the relative growth rate was significantly higher than that of the drought group (≤45% moisture). This strong adaptive capacity under specific water conditions, combined with its dehydration tolerance, suggests that *M. aquaticum* could potentially have a competitive advantage over native submerged plants that rely on stable water bodies, particularly in hydrologically fluctuating habitats. This study revealed that morpho-physiological plasticity driven by water gradients may be a key mechanism contributing to the invasive potential of *M. aquaticum*, providing new insights into its possible expansion potential in zones with fluctuating water levels.

## 1. Introduction

Substrate moisture content critically determines plant growth performance. Plant species exhibit distinct drought tolerances; while some can maintain normal physiological functions under low-moisture conditions, most require adequate moisture for optimal growth [1]. The vulnerability of submerged plants is particularly pronounced during drought exposure in non-native aquatic habitats (e.g., irrigation channels, ponds, and rice paddies) because water deficits can rapidly induce severe physiological dysfunction or even mortality. Notably, invasive aquatic plants that exhibit strong adaptive plasticity and colonization capacity have been reported to establish monoculture communities in non-native habitats, posing substantial ecological threats to existing ecosystems [2]. As an exotic species with invasive potential, *Myriophyllum aquaticum* exhibits amphibious characteristics, enabling its survival in submerged, moist substrates or shallow water environments [3]. The capacity to respond to moisture stress is crucial for adapting to environmental heterogeneity.

Numerous studies have demonstrated that the substrate moisture content critically influences plant physiological and ecological processes. Low-moisture conditions not only induce soil compaction but also inhibit seed germination, new branch development, stem elongation, and root proliferation while disrupting plant–water interactions [4,5], profoundly affecting biomass allocation patterns. Padilla et al. investigated the relationship between plant roots and soil moisture content and found a strong positive correlation between seedling survival and soil moisture. This finding indicates the existence of a soil moisture threshold that governs plant growth [6]. Lower water availability markedly reduces total biomass, aboveground biomass (AGB), belowground biomass (BGB), tiller number, and leaf water content in plants such as clover and ryegrass [7,8], and it impairs transpiration, thereby inducing a cascade of physiological damage, including reduced cell turgor pressure and relative water content [9]. In response to drought stress, plants exhibit diverse morphological, structural, and physiological adaptations [10,11], including increasing stomatal resistance to minimize water loss, developing deep and extensive root systems to enhance water uptake, and forming smaller or succulent leaves to reduce transpiration losses [12]. Existing studies further indicate that invasive aquatic plants often gain competitive advantages through allelopathy [13] (e.g., phenolic compound enrichment and heavy metal-mediated suppression of co-occurring species by Crocus sativus [14]) and enhanced resource-utilization strategies. The formation of monospecific stands of invasive plants reduces habitat heterogeneity, leading to a decline in genetic diversity within native plant populations and potentially facilitating the emergence of more invasive hybrid progeny [15]. Although previous studies have reported the responses of *Myriophyllum aquaticum* to water level fluctuations and drawdown events [16,17], most of them focused on water depth or short-term drying. The present study examines the growth and physiological responses of this species to different substrate moisture contents (0–75%) without surface water, providing new information on its moisture thresholds and adaptive strategies.

Based on this foundation, we established a gradient simulation experiment with substrate moisture content as the key variable to elucidate the adaptive responses (e.g., root architecture, biomass allocation, and key physiological growth indicators) of *M. aquaticum* to varying moisture levels. We hypothesize that: (1) under high substrate moisture (60–75%), rapid root extension and stem node proliferation enable efficient water capture, canopy reconstruction, and enhanced biomass accumulation; (2) under low-to-moderate moisture stress (≤45%), survival strategies such as biomass redistribution are activated but cause physiological damage and growth suppression; and (3) a critical moisture threshold exists for survival, below which mortality occurs, while near-threshold conditions sustain survival only through biomass attenuation. Our findings provide insights into the hydrological adaptation strategies and invasive potential of *M. aquaticum*, thereby supporting ecological risk assessments and targeted control of invasive aquatic plants.

## 2. Results

### 2.1. Growth Characteristics of M. aquaticum Under Different Substrate Moisture Contents

#### 2.1.1. Plant Height

**Figure 1:**
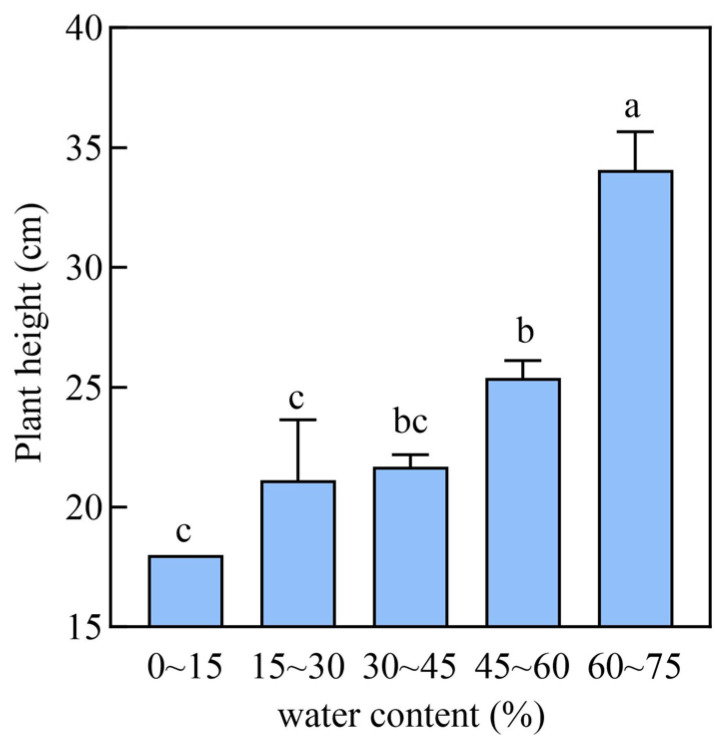
Effect of substrate moisture content on plant height of *Myriophyllum aquaticum*. Different lowercase letters indicate significant differences among treatments (one-way ANOVA followed by Tukey’s test, *p* < 0.05).

Significant differences in the growth performance of *M. aquaticum* were observed among different moisture content treatments (Figure 1). There was a significant difference in plant height at the end of the experiment (*p* < 0.05). Plants in the 0–15% moisture group withered and died by Day 6. Plants in the 60–75% moisture group exhibited the best growth throughout the experiment, and their average height was approximately 1.5 times that of plants in the other surviving treatment groups.

#### 2.1.2. Number of Stem Nodes

**Figure 2:**
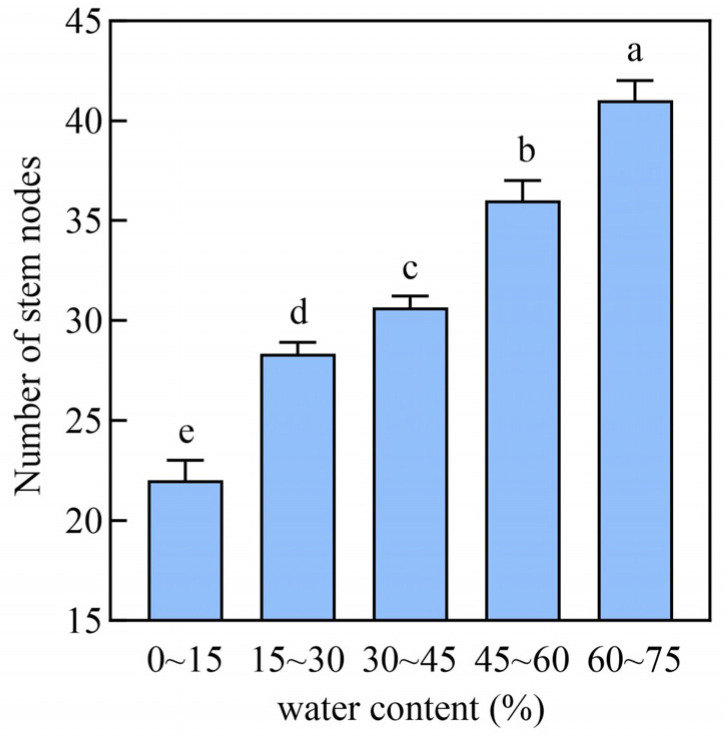
Effect of substrate moisture content on the number of stem nodes of *M. aquaticum*. Different lowercase letters indicate significant differences among treatments (one-way ANOVA followed by Tukey’s test, *p* < 0.05).

Significant differences in stem node numbers were observed among the treatment groups at the end of the experiment (*p* < 0.05). The stem node count increased with increasing moisture content, and the 60–75% moisture group exhibited the highest values. Although stem node counts showed minimal variation among the groups initially, significant differences were clearly present by Day 30 (*p* < 0.05; Figure 2).

#### 2.1.3. Root Length

**Figure 3:**
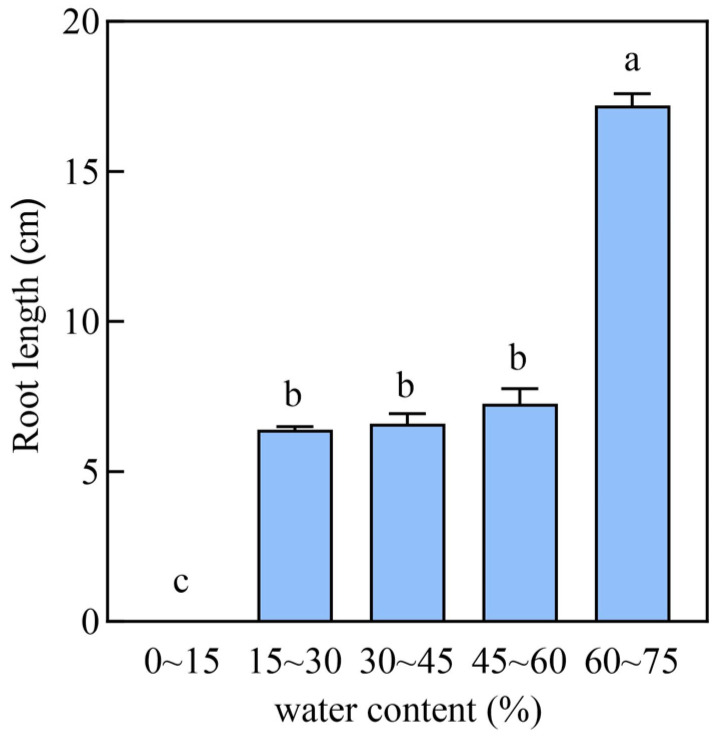
Effect of substrate moisture content on root length of *M. aquaticum*. Different lowercase letters indicate significant differences among treatments (one-way ANOVA followed by Tukey’s test, *p* < 0.05).

Drought stress intensity significantly affected root length (Figure 3), with root length decreasing progressively as stress intensity increased. Notably, *M. aquaticum* in the 0–15% moisture group produced no roots during the experiment. No significant differences were observed in root length among the 15–30% and 45–60% moisture groups (*p* > 0.05), whereas roots were significantly longer in the 60–75% moisture group than in all other groups (*p* < 0.05).

#### 2.1.4. Stem Diameter

**Figure 4:**
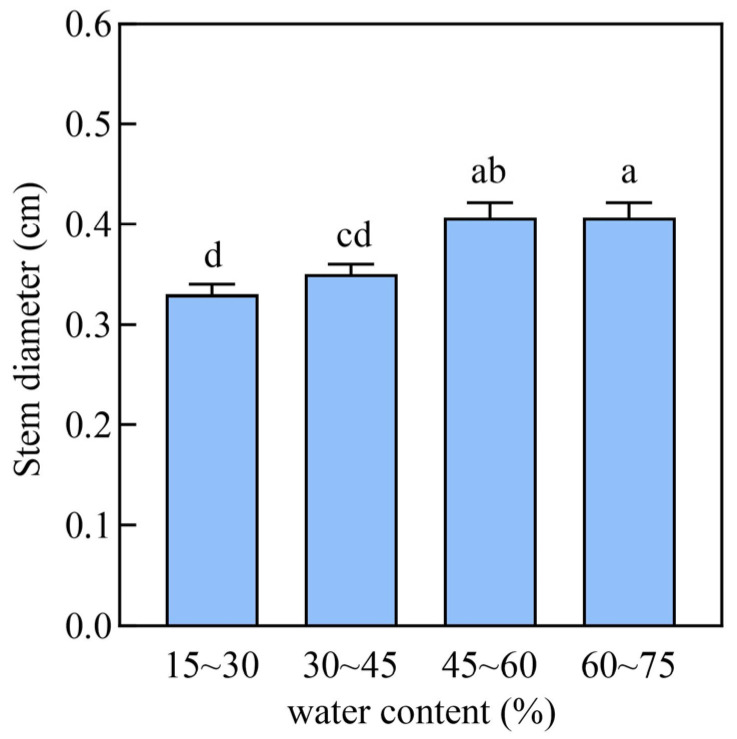
Effect of substrate moisture content on stem diameter of *M. aquaticum*. Different lowercase letters indicate significant differences among treatments (one-way ANOVA followed by Tukey’s test, *p* < 0.05).

The stem diameter of *M. aquaticum* exhibited a decreasing trend with the progressive increase in stress intensity (Figure 4). The most severely stressed treatment group (0–15% moisture) showed the smallest stem diameter (0.33 ± 0.01 cm). Statistical analysis confirmed that there were significantly larger diameters (*p* < 0.05) in the 60–75% moisture group than in the 15–30% moisture group.

#### 2.1.5. Branches and Tillers

**Figure 5:**
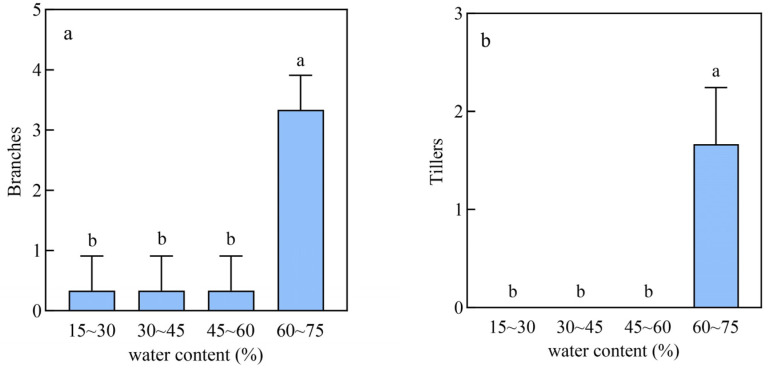
Effect of substrate moisture content on the branching and tillering capacities of *M. aquaticum*, (**a**) branching; (**b**) tillering. Different lowercase letters indicate significant differences among treatments (one-way ANOVA followed by Tukey’s test, *p* < 0.05).

The branching and tillering capacities declined progressively with increasing stress intensity (Figure 5). The number of branches was reduced in the 15–30% and 45–60% moisture groups, showing significantly lower counts than the 60–75% moisture group (*p* < 0.05). Notably, *M. aquaticum* developed no tillers when the substrate moisture content fell below 60%. In contrast, the 60–75% moisture group maintained normal tiller development throughout the experimental period.

#### 2.1.6. Biomass

**Figure 6:**
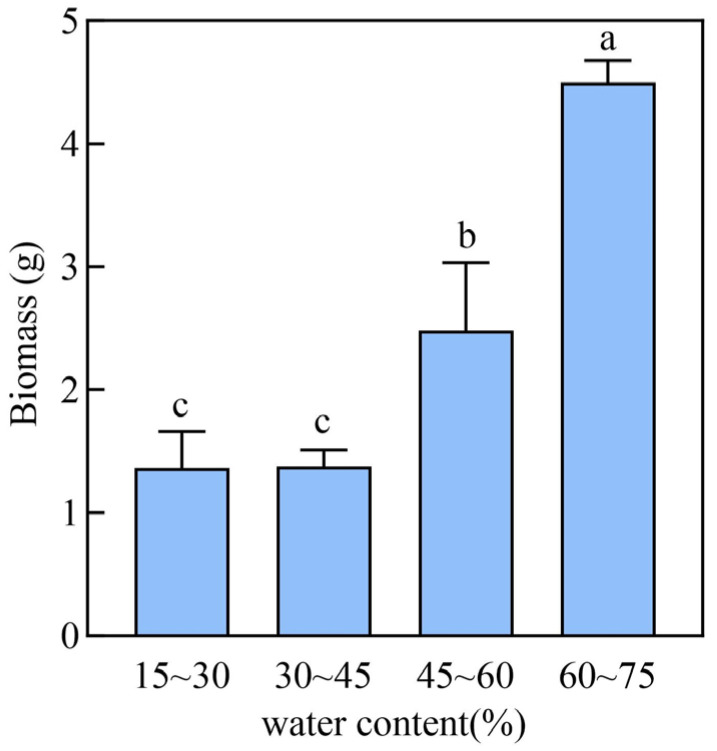
Effect of substrate moisture content on the biomass of *M. aquaticum*. Different lowercase letters indicate significant differences among treatments (one-way ANOVA followed by Tukey’s test, *p* < 0.05).

The biomass of *M. aquaticum* generally decreased with decreasing substrate moisture content. The minimum biomass observed was 1.36 g (15–30% moisture group) at the end of the experiment (Figure 6). At the end of the experiment, the biomass showed no significant differences between the 15–30% and 30–45% moisture groups (*p* > 0.05), although the values in the 15–30% group were marginally lower than those in the 30–45% group.

#### 2.1.7. RGR and AGB:BGB Ratio

The RGR of *M. aquaticum* was significantly influenced by substrate moisture content. In both the 15–30% and 30–45% moisture groups, the RGR values were negative, indicating biomass decline. Notably, the magnitude of the negative RGR was significantly greater in the 15–30% group than in the 30–45% group (*p* < 0.05), demonstrating more severe growth inhibition under intense drought stress.

**Figure 7:**
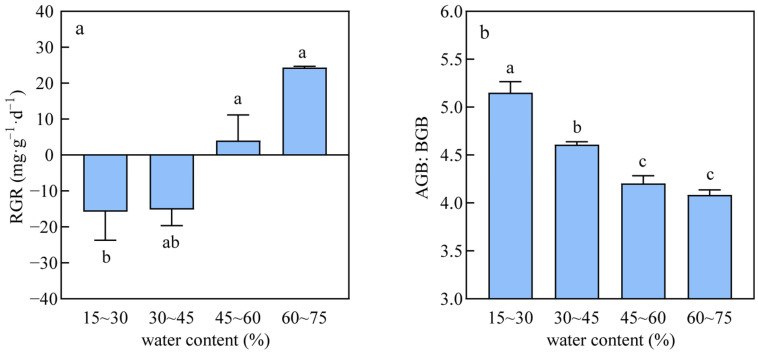
Effect of substrate moisture content on relative growth rate (RGR) (**a**) and aboveground biomass (AGB):belowground biomass (BGB) ratio (**b**) of *M. aquaticum*. Different lowercase letters indicate significant differences among treatments (one-way ANOVA followed by Tukey’s test, *p* < 0.05).

Concurrently, the AGB:BGB ratio exhibited an inverse relationship with substrate moisture (Figure 7). This ratio increased as moisture decreased and decreased as moisture increased.

#### 2.1.8. Total Length of Green and Senescent Internodes

**Figure 8:**
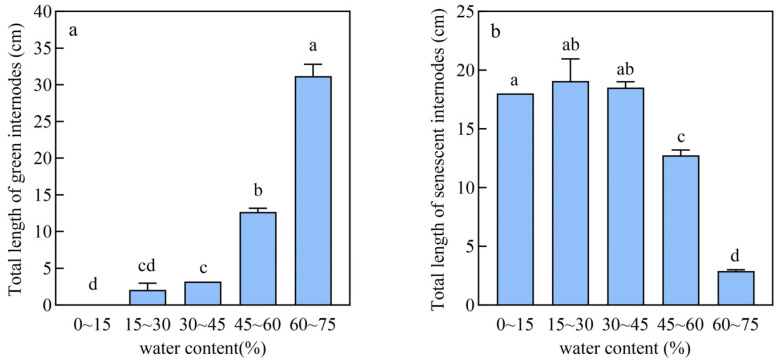
Effect of substrate moisture content on the total length of green and senescent internodes of *M. aquaticum, *(**a**) total length of green internodes; (**b**) total length of senescent internodes. Different lowercase letters indicate significant differences among treatments (one-way ANOVA followed by Tukey’s test, *p* < 0.05).

With decreasing substrate moisture content, the total length of senescent internodes gradually increased. The total length of the senescent internodes in the 0–15% moisture group was significantly greater than that in the 45–75% moisture group (*p* < 0.05). Furthermore, plants in the 0–15% moisture group became dry and had no green leaves by Day 6 of the experiment (Figure 8). Conversely, the total length of green internodes was significantly greater (*p* < 0.05) in the 60–75% moisture group than in the other groups.

### 2.2. Physiological Characteristics of M. aquaticum Under Different Substrate Moisture Contents

#### 2.2.1. Chl-a Content

**Figure 9:**
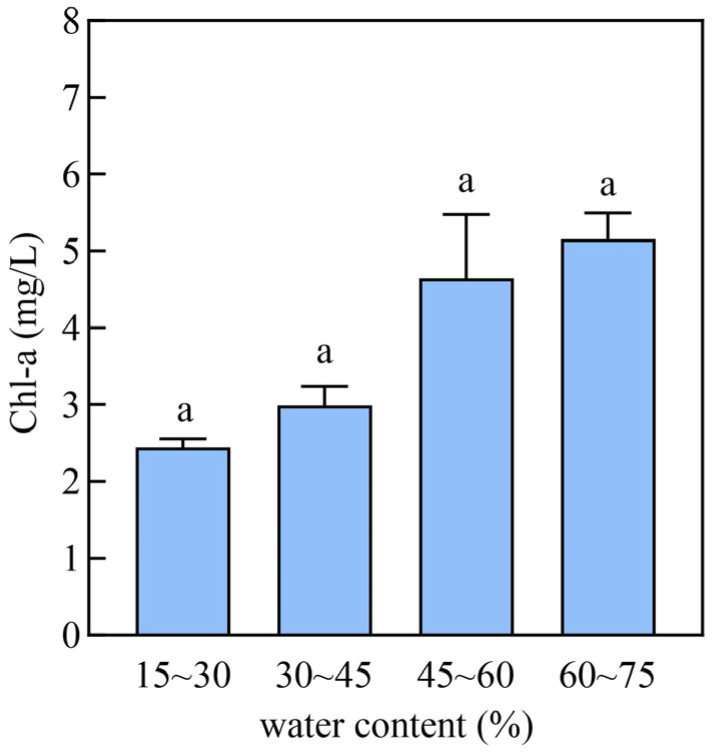
Effect of substrate moisture content on Chl-a content of *M. aquaticum*. Different lowercase letters indicate significant differences among treatments (one-way ANOVA followed by Tukey’s test, *p* < 0.05).

During the experiment, the Chl-a content in the leaves gradually increased with increasing substrate moisture content. At the end of the experiment, the Chl-a content under 60–75% moisture reached a peak value of 3.99 ± 0.5 mg/L. This was approximately 1.6-fold higher than that under 15–30% moisture, 1.4-fold higher than that under 30–45% moisture, and 1.01-fold higher than that under 45–60% moisture (Figure 9). Although the Chl-a content showed an increasing trend with higher moisture levels, the differences among treatments were not statistically significant (*p* > 0.05).

#### 2.2.2. MDA Content

**Figure 10:**
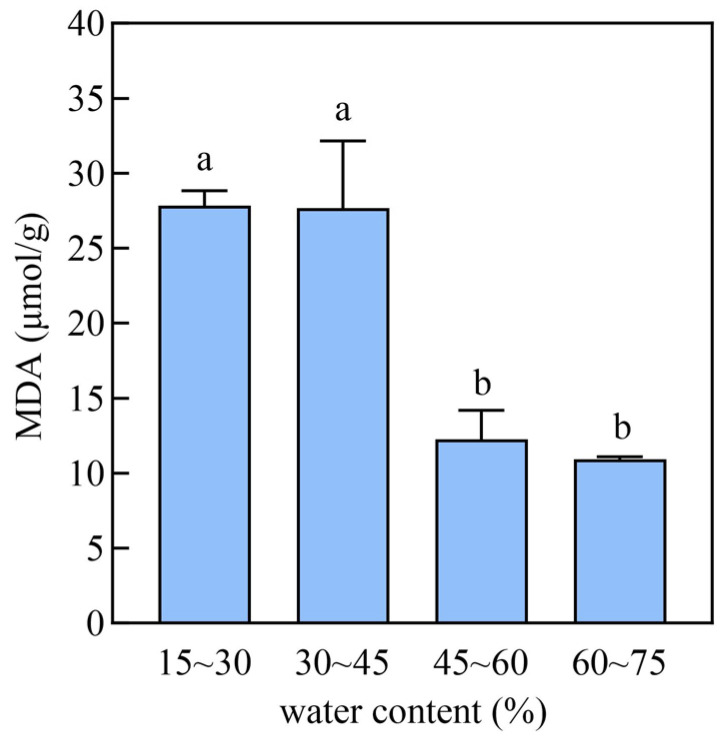
Effect of substrate moisture content on MDA content of *M. aquaticum*. Different lowercase letters indicate significant differences among treatments (one-way ANOVA followed by Tukey’s test, *p* < 0.05).

Drought stress intensity significantly affected the MDA content in surviving *M. aquaticum* leaves (Figure 10). At the end of the experiment, the highest MDA content (27.85 ± 0.98 μmol/g) was observed in the 15–30% moisture group, which was significantly higher (*p* < 0.05) than that in both the moderate- (45–60%) and high-moisture (60–75%) groups. Specifically, the MDA content in the 15–30% group was approximately 1.33-fold and 1.6-fold greater than that in the 45–60% and 60–75% moisture groups, respectively.

Additionally, the final MDA content across all treatments reflected the influence of moisture levels, with more severe drought stress leading to markedly higher accumulation of MDA, while higher moisture conditions resulted in comparatively lower levels.

### 2.3. Overall Effect of Substrate Moisture Content on M. aquaticum

The ANOVA results revealed a significant effect of substrate moisture content on multiple growth, morphological, and physiological indices of *M. aquaticum* (*p* ≤ 0.0004 for all indices). Effect size analysis (η^2^) revealed significant variation in moisture sensitivity across indicators (Table 1). Root length, total green internode length, stem node number, and total senescent internode length showed the highest sensitivity (here, “sensitivity” refers to the effect size (η^2^) derived from the ANOVA results), indicating that moisture decisively regulates plant architecture (e.g., root system development, internode elongation, and nodal proliferation). The AGB:BGB ratio, plant height, and total biomass were also strongly influenced, reflecting the critical role of moisture in overall growth rates and resource-allocation patterns. In contrast, moisture exerted relatively weaker (although statistically significant) effects on stem diameter, tiller number, Chl-a content, relative growth rate, MDA level, and branch number.

**Table 1:** Strength ranking of the effect of substrate moisture content on growth indices of *M. aquaticum*.

Indicator	η^2^	*p*	Significance Ranking
Root length	0.9976	*p* < 0.0001	1
Total length of green internodes	0.9960	*p* < 0.0001	2
Number of stem nodes	0.9886	*p* < 0.0001	3
Total length of senescent internodes	0.9856	*p* < 0.0001	4
AGB:BGB	0.9785	*p* < 0.0001	5
Plant height	0.9592	*p* < 0.0001	6
Biomass	0.9564	*p* < 0.0001	7
MDA	0.9332	*p* < 0.0001	8
RGR	0.9247	*p* < 0.0001	9
Stem diameter	0.9122	*p* = 0.0001	10
Number of tillers	0.9036	*p* = 0.0002	11
Chl-a	0.9035	*p* < 0.0001	12
Number of branches	0.8835	*p* = 0.0004	13

### 2.4. Principal Component Analysis of Different Traits of M. aquaticum

Principal component analysis (PCA) was performed on 13 growth and physiological traits. The KMO value was 0.80, and Bartlett’s test of sphericity was significant (χ^2^ = 1862.36, *p* < 0.001), confirming the suitability of the data for PCA.

Two principal components with eigenvalues > 1 were extracted, which together explained 94.90% of the total variance (PC1: 84.63%, PC2: 10.27%; Table 2). PC1, which accounted for the majority of the variance, represented a clear gradient of growth performance versus drought stress. It showed strong positive loadings on growth-related traits (plant height, stem node number, root length, biomass, RGR, total green internode length, branch number, tiller number, and Chl-a) and strong negative loadings on stress-related traits (total senescent internode length, AGB:BGB ratio, and MDA content). Thus, samples from the high-moisture treatment (60–75%) had high PC1 scores, while those from low-moisture treatments had low (negative) PC1 scores. PC2 captured the remaining variation (detailed loadings shown in Table 2).

Overall, the PCA results indicate that substrate moisture content strongly coordinates changes across multiple morphological and physiological traits in *M. aquaticum*.

## 3. Discussion

### 3.1. Influence of Substrate Moisture Content on M. aquaticum Morphological Characteristics and Biomass

Substrate water stress affects all plant organs. Under suboptimal moisture conditions, plants initiate physiological and biochemical responses that manifest as stunted height and reduced stem diameter [18]. As water stress intensified, M. aquaticum plant height gradually decreased, whereas stem diameter thinned progressively, with intergroup differences becoming more pronounced over time. Throughout the experiment, the 60–75% moisture group maintained optimal growth conditions. In contrast, the 0–15% group succumbed to acute drought conditions, and the 15–30% group exhibited stunted growth.

Plant biomass accumulation is crucial for individual growth and development [19,20], and water availability is a key regulator of biomass partitioning [21,22,23]. The experiment revealed significant heterogeneity (*p* < 0.05) in the biomass-allocation patterns of *M. aquaticum* across the substrate moisture gradient. Reduced water stress triggered increased total biomass accumulation and a higher proportional allocation to aboveground dry matter. Biomass accumulation peaked in the 60–75% moisture group, where the RGR also reached its maximum. Increased water stress significantly reduced *M. aquaticum* BGB. Critically, BGB accumulation is strongly correlated with aboveground production efficiency. At 60–75% moisture, the root allocation strategy demonstrated optimal adaptability, enabling efficient aboveground resource utilization. While plants in the mild-stress groups showed slow biomass gains over time [24], *M. aquaticum* in the 15–30% and 30–45% groups exhibited progressive biomass reduction under prolonged stress. This aligns with the findings of Liu et al. on Bothriochloa ischaemum drought responses [25], confirming species-specific patterns across stress intensities and durations.

Drought, a primary threat to plants, accelerates leaf senescence [26,27]. In *M. aquaticum*, senescence manifests as basipetal drying (from the substrate contact points upward). Only the 60–75% moisture group remained largely unaffected; the other groups exhibited progressive drying with increasing stress duration. At the end of the experiment, the final drying severity was statistically comparable between the 0–15% and 30–45% groups (*p* > 0.05). In *M. aquaticum*, intensifying water stress progressively reduced the branch number, tiller production, and root length. Notably, tillering ceased when the moisture content was below 60%. This reflects a drought adaptation strategy in which plants reduce energy allocation to roots and branches and prioritize resource investment in the main stem to maximize photosynthetic survival.

### 3.2. Effects of Substrate Moisture Content on M. aquaticum Physiological Characteristics

The Chl-a content in *M. aquaticum* tended to increase with higher substrate moisture availability, although the differences among treatments were not statistically significant (*p* > 0.05). This trend is consistent with previous findings that insufficient substrate moisture can impair chlorophyll synthesis [28]. Water deficiency may induce stomatal closure, limit CO_2_ diffusion, suppress the synthesis of light-harvesting complexes, and promote reactive oxygen species accumulation, all of which can affect chlorophyll levels [29,30,31]. Therefore, the observed decreasing trend in Chl-a content under lower moisture conditions represents a physiological response of *M. aquaticum* to water stress, even though it did not reach statistical significance in this study.

As the primary product of membrane lipid peroxidation, MDA serves as a direct biomarker of oxidative damage to cellular membranes [32,33]. Consistent with the findings of Gu et al. on drought-induced MDA accumulation in plants [34], all treatments exhibited progressive MDA increases under sustained stress. This persistent accumulation signifies critical physiological constraints. While *M. aquaticum* demonstrates limited short-term stress tolerance, its capacity to resist prolonged or severe drought is physiologically constrained. Chronically elevated MDA levels reflect the progressive degradation of membrane integrity and metabolic dysfunction, ultimately compromising plant viability, as evidenced by mortality in the 0–15% moisture group by Day 6.

### 3.3. Invasive Potential of M. aquaticum from the Perspective of Substrate Moisture Content

The global expansion of *M. aquaticum* is intrinsically linked to its adaptability to substrate moisture gradients. This study extends previous research that mainly focused on water level fluctuations and drawdown events [16,17] by examining its responses under a controlled substrate moisture gradient (0–75%) without surface water. Analysis of variance (ANOVA) and principal component analysis indicated that substrate moisture content is an important regulatory factor for the growth and physiological processes of *M. aquaticum*.

The species exhibited optimal growth at 60–75% substrate moisture content. Under this condition, plants achieved approximately 1.5-fold greater height compared with drought conditions (≤45% moisture), along with significantly enhanced stem node production, root elongation, and biomass accumulation (*p* < 0.05). This moisture range also promoted vigorous branching and vegetative growth.

Furthermore, *M. aquaticum* exhibits notable drought resilience at ≤45% moisture. At 15–30% substrate moisture, it activated multiple adaptive responses: morphologically, root shortening maintained water uptake while senescent internode length increased by about 30% compared to high-moisture groups, forming transpiration-reducing barriers [35]; allocationally, biomass allocation shifted toward aboveground parts (elevated AGB:BGB ratio); and physiologically, metabolic regulation occurred despite elevated membrane damage (MDA ≈ 27.85 μmol/g, ~1.6 times higher than in the high-moisture group). This adaptive suite enabled survival for more than 30 days during drought periods, whereas many native submerged species (e.g., Vallisneria natans and Potamogeton crispus) tend to experience rapid biomass decline under comparable stress [36].

When the sediment surface dried but subsurface moisture remained at 60–75%, the plants maintained efficient root water uptake and rapid canopy regeneration, resulting in higher biomass accumulation rates (more than 1.5-fold) compared to the drought groups within 30 days.

These findings suggest that the phenotypic plasticity of *M. aquaticum* in response to substrate moisture gradients may facilitate its persistence in hydrologically fluctuating habitats. However, since this study did not include direct competition experiments with native species, statements regarding competitive displacement, light-resource monopolization, and formation of monospecific communities should be viewed as potential outcomes that require further experimental validation.

## 4. Materials and Methods

### 4.1. Experimental Design

**Figure 11:**
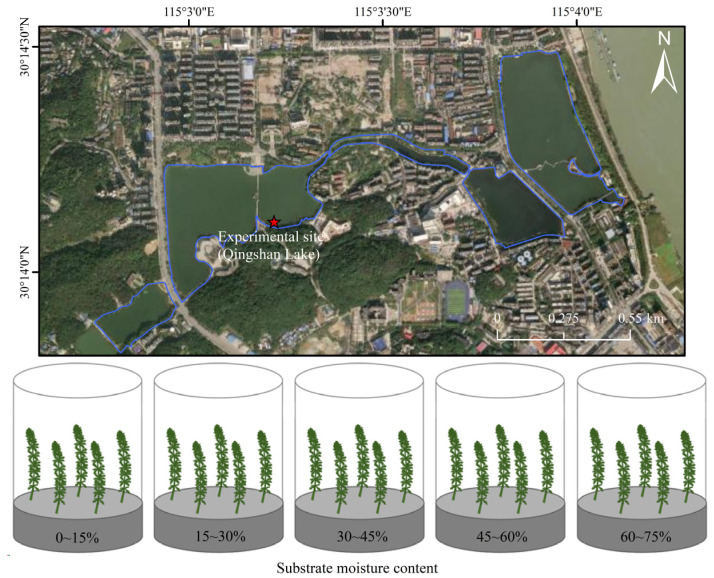
Schematic diagram of the experiment.

This study was conducted in June 2024 along the shoreline of Qingshan Lake (115°3′17.02″ E, 30°14′6.70″ N), Huangshi City, Hubei Province, under natural outdoor conditions (Figure 11). During the experimental period, the average daytime temperature ranged from 26 to 32 °C, nighttime temperature from 20 to 24 °C, and relative humidity from 70% to 90%. Natural sunlight provided the light conditions, with a photoperiod of approximately 14 h.

Uniformly developed *M. aquaticum* individuals (mean height: 15.0 ± 0.5 cm) were transplanted into polyethylene pots containing 10 cm of lake bottom sediment. The sediment was sieved through a 10-mesh screen (2 mm) to remove shells and gastropods, followed by 7 days of pre-cultivation. Five plants were arranged circularly in each pot. A substrate moisture gradient was established with five levels: 0–15%, 15–30%, 30–45%, 45–60%, and 60–75% (n = 4 pots per treatment). The 60–75% level corresponds to the measured moisture content of the Qingshan Lake sediment, representing the optimal condition for *M. aquaticum*. Lower levels were obtained by gradual dehydration from this baseline.

To maintain the target substrate moisture levels, pots were weighed daily using an electronic balance, and deionized water was added accordingly. Soil moisture was periodically verified with a portable soil moisture meter (MQ-SCTR2 handheld soil moisture meter, Mingqiao, Zibo, Shandong, China). All pots were randomly arranged on the lakeshore platform and re-randomized every 5 days to minimize positional effects. At each sampling time (every 10 days), one plant was randomly harvested from each pot for measurements, while the remaining plants continued to grow in the same pot.

### 4.2. Indicator Measurement

During the 30 d experimental period, the growth indicators of *M. aquaticum* were measured every 10 d. We randomly selected six plants from each treatment group, rinsed them with water after harvesting to remove adsorbed impurities on the surface of the plant, drained them, and measured the plant height, number of stem nodes, root length, stem diameter, number of branches, number of tillers, total biomass, AGB, BGB, and internode length. We used an analytical balance to measure the fresh weight, which was used to calculate the relative growth rate (RGR) as follows [37]:

(1) RGR = ln(Wf/Wi)/D

where Wi and Wf represent the initial and final fresh weights, respectively, and D denotes the experimental duration in days.

Chlorophyll a (Chl-a) and malondialdehyde (MDA) were also measured at 10 d intervals. Specifically, MDA was quantified using the thiobarbituric acid method [38,39], while Chl-a was determined via ethanol extraction spectrophotometry [40]. The details are provided below.

Following harvesting, *M. aquaticum* branches were rinsed and transported to the laboratory to undergo 24 h dark adaptation. Intact apical leaves (3–5 per plant) were then excised from the main stems, with 0.2 g (fresh weight) subsamples used for Chl-a quantification and 0.5 g subsamples for MDA determination.

We measured the MDA content using the thiobarbituric acid method with pure water as a blank. The absorbance values were determined at 450 nm, 532 nm, and 600 nm, and the MDA content was calculated using the following equation [41,42]:

(2) A532 − A600 = 15,500 × C × L

(3) C (μmol/L) = 6.45(A_532_ − A_600_) − 0.56A_450_

(4) $$MDA(\mu mol/g)=\frac{C\times V}{W\times 1000}$$

where A_450_, A_532_, and A_600_ represent the absorbance values at 450 nm, 532 nm, and 600 nm, respectively, and L denotes the cuvette pathlength (cm). C is the MDA concentration in the extract (μmol/L), V is the total volume of the extract (mL), and W is the sample fresh weight (g). The factor 1000 converts microliters to milliliters. The MDA concentration in the plant extracts was calculated using this equation, enabling the quantification of the tissue MDA content.

The Chl-a content was determined via ethanol extraction spectrophotometry with 95% ethanol as a blank, and absorbance was measured at 665 nm, 649 nm, and 470 nm. The Chl-a content was calculated using the following formula [43,44]:

(5) Chl-a = 13.95 × A_665_ − 6.88 × A_649_

where A_649_ and A_665_ are the absorbance values at 649 nm and 665 nm, respectively.

### 4.3. Data Analysis

Statistical analyses were performed using Excel 2019 and GraphPad Prism 10.1.2. Each treatment had four replicate pots (n = 4). To avoid pseudoreplication, one plant was randomly selected and harvested from each pot at each sampling time for measurements. Thus, all statistical analyses were based on the four independent pots as the true experimental units (n = 4 per treatment). One-way analysis of variance (ANOVA) was used to test the effects of substrate moisture content on growth and physiological parameters, followed by Tukey’s HSD test for multiple comparisons (*p* < 0.05). Effect sizes (η^2^) were calculated from the ANOVA results. Geographic visualization was implemented in ArcGIS 10.2 to generate the study area map.

## 5. Conclusions

Through a simulation experiment, we found that substrate moisture content significantly affected the physiological growth characteristics of *M. aquaticum*. Under a substrate moisture content of 60–75%, plant height, stem diameter, biomass accumulation, and RGR reached their peak values, accompanied by enhanced branching and tillering abilities. When moisture content dropped below 45%, the plants activated several adaptive mechanisms, including shortened roots, increased senescent internode length, a shift toward aboveground biomass allocation, a decreasing trend in Chl-a content, and elevated MDA accumulation. Plants survived under 15–30% moisture conditions despite continuous biomass decline, whereas plants died when the moisture content was ≤15%. Notably, even without surface water, substrate moisture levels of 60–75% still supported efficient root water uptake and rapid canopy regeneration, resulting in biomass accumulation rates more than 1.5 times higher than those of drought groups. These findings suggest that strong physiological plasticity and tolerance to fluctuating moisture conditions may contribute to the invasive potential of *M. aquaticum* in periodic wet–dry habitats, providing important insights for the ecological management of invasive aquatic plants.

## Figures and Tables

**Table 2:** Principal component loadings for physiological and growth indicators of *M. aquaticum*.

Physiological Growth Indicators	PC1 Loading	PC2 Loading	Contribution Direction
AGB/BGB	−0.857		↓
Plant height	0.955		↑
Number of stem nodes	0.981		↑
Stem diameter	0.827	−0.524	↑
Number of branches	0.855		↑
Number of tillers	0.849		↑
Root length	0.918		↑
Biomass	0.975		↑
RGR	0.968		↑
Total length of green internodes	0.989		↑
Total length of senescent internodes	−0.988		↓
Chl-a	0.904		↑
MDA	−0.869		↓
Eigenvalue	11.002	1.335	
Variance contribution	84.633	10.271	
accumulate %	84.633	94.904	

Note: ↑ indicates strong growth vitality and low stress level; ↓ indicates that growth is inhibited and the degree of stress is high.

## Data Availability

The original contributions presented in this study are included in the article; further inquiries can be directed to the corresponding author.
